# Loss of ovarian hormones is detrimental in early disease stages of mouse models of Alzheimer’s disease and multi-etiology dementia

**DOI:** 10.1186/s13293-025-00795-4

**Published:** 2025-12-05

**Authors:** Charly Abi-Ghanem, Alex K. Opiela, Aaron S. Paul, McKenzie L. Comito, Lawrence Hao, Grace Martino, Nyi-Rein Kyaw, Abigail E. Salinero, Febronia M. Mansour, Richard D. Kelly, Ann M. Mutahi, Avi Sura, Christina A. Thrasher, Emily A. Groom, Molly R. Batchelder, Kristen L. Zuloaga

**Affiliations:** 1 Department of Neuroscience & Experimental Therapeutics, Albany Medical College, 47 New Scotland Avenue; MC-136, Albany, NY 12208 USA; 2 Behavioral Neuroscience Program, Department of Psychology, Binghamton University, 4400 Vestal Parkway E, Binghamton, NY 13902 USA

**Keywords:** Alzheimer’s disease, Multi-etiology dementia, Menopause, Estrogen, Ovariectomy, Cognitive decline

## Abstract

**Background:**

Up to 80% of Alzheimer’s disease (AD) patients suffer from brain vascular damage resulting in multi-etiology dementia (MED). Sex is a well-known risk factor for dementia; out of three AD patients two are women. 17β-estradiol, a predominant ovarian hormone in woman before menopause, is known to have beneficial effects on the cerebrovasculature, neuroinflammation and neuroprotection. Here, we investigated the consequences of the loss of ovarian hormones caused by surgical menopause (ovariectomy) on AD and MED.

**Methods:**

The App^NL−F^ knock-in mice were used to model AD. At about 5.5 months of age, a stage corresponding to early disease pathology, female App^NL−F^ mice were subjected to ovariectomy (OVX) or sham surgery (Intact) and left to recover for 3 weeks to clear any endogenous gonadal hormones. In half of the mice from each group, MED was modeled using chronic cerebral hypoperfusion (unilateral carotid artery occlusion), a model of vascular contributions to cognitive impairment and dementia (VCID). Control animals (AD only model) received sham surgery. Mice were then subjected to a battery of behavioral tests before being euthanized and brains were collected to assess pathology.

**Results:**

We found that loss of ovarian hormones impairs spatial learning and memory, impairs activities of daily living, and affects underlying pathology including compromising microglial response. Some of these effects were exacerbated by cerebral hypoperfusion (VCID).

**Conclusions:**

These results shed light on the effects of ovarian hormone loss after surgical menopause in female mouse model of AD and MED in order to better understand sex-specific risk factors.

**Supplementary Information:**

The online version contains supplementary material available at 10.1186/s13293-025-00795-4.

## Abstract (plain language summary)

Alzheimer’s disease (AD) is the most common form of dementia. AD affects more women than men. Most AD patients do not have pure AD, instead they have a mix of AD and other forms of dementia. This mix of multiple dementia types is known as multi-etiology dementia (MED). Vascular dementia, caused by damage to blood vessels, is the most common type of dementia that occurs with AD in MED. Since 2/3 of those with AD are women, we wanted to determine how menopause impacts AD and MED. 17β-estradiol is a prominent hormone produced in the ovaries of women before menopause, but it is lost following menopause. 17β-estradiol is known to have multiple beneficial effects on the brain and the vasculature. Here, we used a genetic mouse model of AD combined with a surgery to reduce blood flow to the brain to model MED. To model loss of ovarian hormones during menopause, we removed the ovaries. We found that menopause caused impairments in some types of learning, memory, and the ability to perform some normal daily activities. Menopause also causes changes in the brain that resulted in worse dementia pathology. Some changes were even more severe in the MED mice compared to AD mice. These results indicate that hormones produced by the ovaries that are lost during menopause are important and protective in models of AD and MED. These findings are important as they will help in future studies for diagnosing and guiding treatment of dementia in female patients.

**Supplementary Information:**

The online version contains supplementary material available at 10.1186/s13293-025-00795-4.

## Highlights

In mouse models of AD and MED, at a stage that corresponds to early disease pathology, loss of ovarian hormones leads to:

Impaired spatial learning and memory

Impaired activities of daily living

Increased insoluble Aβ levels and astrogliosis in the hippocampus

Impaired microglia response across several brain regions

**Supplementary Information:**

The online version contains supplementary material available at 10.1186/s13293-025-00795-4.

## Background

Patients diagnosed with Alzheimer’s disease (AD), the most common form of dementia, often present with changes in their brains related to at least one other cause of dementia, most commonly vascular damage. This is known as mixed or multi-etiology dementia (MED) [1]. Cerebrovascular pathology, which is often found in AD brains, leads to cerebral blood flow deficits which can exacerbate damage and contribute to cognitive impairments. In fact, cerebrovascular pathology underlies the second most common form of dementia known as vascular contributions to cognitive impairment and dementia (VCID) [2–4]. AD affects women more than men (2:1 ratio), which highlights the need to explore sex-specific risk factors related to disease pathology and outcomes [1, 5].

A key sex-specific risk factor for dementia is ovarian hormone loss associated with menopause [6, 7]. A major indicator of the involvement of ovarian hormones is the association between menopause and the increased risk of dementia [5, 8–10]. Several studies have associated an earlier age at menopause (surgical or natural) with increased cognitive decline [11–13]. Further, recent studies have shown that younger age at menopause onset is associated with increased AD pathology and worse disease progression [14–18]. Menopause, whether naturally occurring or surgically induced (ovariectomy performed for health-related issues), results in the loss of gonadally produced 17β-estradiol (E2) with low circulating and brain E2 levels and eventually reproductive senescence [19]. Indeed, E2 has been shown to have a plethora of beneficial effects on the nervous system including cognitive, physiological and cerebrovascular processes [9, 20–22]. In women, structural and functional neuroimaging studies implicate both endogenous and exogenous forms of estrogens in shaping brain networks and on verbal episodic and working memory [23–26]. Preclinical studies in rodent models support the beneficial role of estrogens, such as E2, in normal aging and in models of neurodegenerative diseases [22]. Loss of E2 in wild-type (WT) rodent menopause models has been shown to lead to insulin resistance and a rapid progression into the metabolic syndrome [27], increase blood–brain barrier (BBB) permeability [28], impair neurovascular coupling [29], decrease the number of synaptic spines and reduce plasticity [30], modulate neurogenesis [31], and induce changes in microglial activation and neuroinflammation [32–34]. Several of these effects are reversed after E2 replacement treatment. In line with these data, our lab has shown that in a VCID mouse model, inducing menopause leads to exacerbated cognitive decline [35]. Further, in an AD mouse model, menopausal mice have worse cognitive decline, and impaired neuroinflammation and neurogenesis on a high fat diet compared to non-menopausal controls [36, 37].

In this study we aimed to examine the effects of the loss of ovarian hormones due to surgical menopause (ovariectomy, OVX) on cognition and pathology in a pure AD and a MED models. Using the knock-in mouse model of AD, App^NL−F^ mice, we modeled MED by inducing cerebral hypoperfusion using a unilateral common carotid artery occlusion, a well-established VCID model. App^NL−F^ mice carry a humanized APP gene with the Swedish and Iberian mutations which results in a slow disease progression with plaques and gliosis starting at 6 month of age and no cognitive impairment until they are 18 mo [38]. This allowed us to study the effects of ovarian hormone loss at a stage corresponding to early prodromal AD as opposed to other transgenic models showing rapid pathology and cognitive decline progression. Comparing gonadally intact mice from both dementia groups to ovariectomized mice allowed us to test whether loss of gonadal hormones would exacerbate either of the dementia types.

## Methods

### Animals and experimental design

**Fig. 1:**
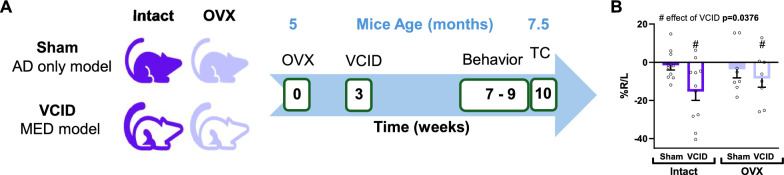
Experimental design (**A**). Female mice at 5 months of age received either an ovariectomy (OVX) or a sham (Intact) surgery. mice were allowed to rest for 3 weeks then received surgery to model VCID which results in a MED model. control animals (AD only model) received sham surgery. one month later, mice were subjected to a battery of behavioral tests then cerebral blood flow was measured before authorization and brains collection. (**B**) at the end of the study, cortical blood flow was measured using laser speckle contrast imaging 6 weeks after UCCAO surgery. unilateral chronic cerebral hypoperfusion was evaluated using the differential between the signals the right and left hemispheres (%R/L) in the temporal subscapular area of the brain. data were analyzed using a 2-way ANOVA. # p < 0.05 effect of VCID; n = 8–11 mice/group. AD: alzheimer’s disease, MED: multi-etiology dementia, OVX: ovariectomy, VCID: vascular contributions to cognitive impairment and dementia, TC: tissue collection

All experiments were approved by the Albany Medical College Animal Care and Use Committee and in compliance with the ARRIVE guidelines. App^NL−F^ (App^tm2.1Tcs^/App^tm2.1Tcs^) mice [38] were acquired congenic on a C57BL/6 J background from Dr. John Cirrito at Washington University following MTA from Riken, Japan. These mice were bred in house and were fed a standard chow diet (Purina Lab Diet 5P76) for the duration of this study. Female mice of about 5 months of age were housed (3–4 per cage) at 21 °C, 30–70% humidity, with a 12 h light/dark cycle. To model surgical menopause, which usually happens in younger women compared to natural menopause which occurs in middle aged ones, cages of mice were randomly selected to receive either an ovariectomy (OVX) or a sham surgery (Intact). Mice were allowed to rest for 3 weeks. Within each group half the cages were subjected to a unilateral carotid artery occlusion surgery (UCCAO), a model of VCID, inducing chronic cerebral hypoperfusion in the right hemisphere to model MED. Control animals (AD only model) received sham surgery. One month later, mice were subjected to a battery of behavioral tests for 2 weeks. At the end of the study (mice ~ 7.5 months old), blood flow measurement was collected using laser speckle imaging as described previously [35, 39, 40]. Mice were then euthanized by deep anesthesia followed by cardiac puncture to collect plasma then perfusion with ice cold heparinized saline. Brains were collected and either post-fixed for IHC or regionally dissected and flash frozen for molecular biology. One mouse in the Intact Sham group presented with a growth on its ovary during tissue collection and was removed from the study. Figure 1A shows the experimental groups and timeline.

### Ovariectomy

Bilateral ovariectomy was performed under isoflurane anesthesia as described previously [41]. Control mice received a sham surgery and were left gonadally intact (Intact group).

### Chronic cerebral hypoperfusion

UCCAO or sham surgeries were performed as described previously [35, 39, 40] 3 weeks after OVX surgeries to allow enough recovery time. Animals were allowed to recover for 4 weeks before behavior testing started.

### Open field testing

Locomotor activity was evaluated using an open field test, as previously described [35, 36, 39–41]. Mice were placed in a square arena (45 × 45 cm) and allowed to explore freely for 10 min, then removed and placed in a “recovery cage” so as not to expose naïve cage mates to them. Videos were analyzed using ANY-maze software (Stoelting, Wood Dale, Illinois, USA). Total distance traveled (m) was measured as a proxy for locomotor activity. Intact Sham n = 13 mice, Intact VCID n = 15 mice, OVX Sham n = 13 mice, OVX VCID n = 14 mice.

### Object place and novel object recognition

Spatial recognition and object recognition memory were tested back-to-back using the combined test as described before [36, 41]. A spatial cue (a horizontal piece of black tape) placed on the wall of the open field arena where objects were initially placed. The test consisted of three 10 min trials: a “training” trial, the object place recognition test (OPRT) trial, and the novel object recognition test (NORT) trial, with an inter-trial interval of one and a half hours. Mice that did not interact with both objects during “training” trial, or those that didn’t interact with the objects for at least 20 s during the 10 min testing were excluded from analysis. For OPRT: Intact Sham n = 9 mice, Intact VCID n = 12 mice, OVX Sham n = 10 mice, OVX VCID n = 9 mice. For NORT: Intact Sham n = 11 mice, Intact VCID n = 13 mice, OVX Sham n = 10 mice, OVX VCID n = 10 mice.

### Barnes maze

The Barnes maze test, used to evaluate hippocampal-dependent spatial learning and memory, was performed using as previously described protocol [35, 36]. Mice were trained to find an escape pod located under one of the 20 holes in the brightly lit maze (Stoelting, Catalog # 60,170), using spatial cues in the room. Mice were given two training trials a day (3 min each or until the mouse found the escape pod) and 3 h between trials during the same day for 4 consecutive days to test spatial learning. Spatial memory was tested on the sixth day (1 day break) by allowing the mice to explore the maze for 2 min. Mice that failed to learn the position of the escape hole were excluded from the memory test analysis (one mouse excluded in each group). Video recordings for the probe trial of some mice were lost due to a computer malfunction resulting in an exclusion of these mice from the memory test (but not learning) analysis. This resulted in the following n numbers: for spatial learning: Intact Sham n = 12 mice, Intact VCID n = 15 mice, OVX Sham n = 13 mice, OVX VCID n = 14 mice; for spatial memory: Intact Sham n = 8 mice, Intact VCID n = 10 mice, OVX Sham n = 9 mice, OVX VCID n = 10 mice.

### Nest building test

The nest building test was used to evaluate performance of activities of daily living as previously described [35, 40, 41]. Mice were provided with intact nestlet material and individually housed overnight with food and water. The next day (16 h later), mice were carefully removed from the cages and returned to their group home cage. The nests were scored by three independent scorers, blinded to the mouse ID and experimental group, using a scale of 1–5 in 0.5-point increments [42], with 1 being the lowest possible score and 5 being the highest. For each mouse the final value was the average of the three scorers. Intact Sham n = 13 mice, Intact VCID n = 15 mice, OVX Sham n = 13 mice, OVX VCID n = 14 mice.

### Aβ quantification using an enzyme-linked immunosorbent assay

As previously described, frozen hippocampi were homogenized in 200 µL of T-Per buffer (78,510, ThermoFisher Scientific) supplemented with protease and phosphatase inhibitor cocktail (HALT, 1,861,284 ThermoFisher Scientific) and spun at 21,000 g for 20 min at 4◦C. The supernatant containing soluble proteins was removed and used to quantify total soluble protein levels using a Bicinchoninic Acid (BCA) protein assay according to manufacturer’s instructions (23,227, ThermoFisher Scientific). The pellet containing insoluble proteins was resuspended in 100 µL of 70% formic acid and stored at −80◦C. On the day of the enzyme-linked immunosorbent assay (ELISA), the samples were thawed, and 2 mL (20x) of neutralizing solution (1 M Tris base, 0.5 M Na2HPO4, 0.05% NaN3) were added. The neutralized sample solution was processed for Aβ quantification using the Human Aβ40 ELISA (KHB3481, ThermoFisher Scientific) and Human Aβ42 ELISA (KHB3441, ThermoFisher Scientific) kits according to the manufacturer’s instructions. Outliers were identified using Grubb’s test and excluded if affecting normality. Exclusions for Aβ40: 1 mouse in the Intact Sham and 1 in the OVX Sham group. Exclusions for Aβ42: 1 mouse in the Intact Sham and 1 in the Intact VCID group.

### Immunofluorescent labeling

Brains used for immunofluorescent labeling were fixed overnight in a 4% paraformaldehyde (PFA) solution, then cryoprotected in 30% sucrose, then frozen in optimal cutting temperature (O.C.T.) solution (23–730-571, ThermoFisher Scientific) and stored at −80 °C until further processing. Using a cryostat (Cm1950, Leica), 35 μm-thick sections were obtained and stored at 4 °C. A 25G needle was used to mark the left side of the brain contralateral to the hypoperfusion side. Immunofluorescent labeling was done as previously described [39, 43, 44]. Briefly, slices were permeabilized and blocked, using a 0.3% PBS with triton (TPBS) and 5% donkey serum solution, for one hour at room temperature. Primary antibodies were applied overnight at 4 °C in blocking solution. After 3 washes in PBS, the corresponding secondary antibodies and DAPI (1:1000; D1306, Thermo Fisher Scientific) were added in blocking buffer for 1 h at room temperature before mounting between slides and coverslip using Fluoromount-G™ mounting medium (0100–01; Southerbiotech). Images of brain slices were obtained at 10 × magnification using the Axio Observer Fluorescent Microscope (Carl Zeiss Microscopy, Oberkochen, Germany).

Antibodies used for the microglia panel: rabbit anti goat anti-Iba-1 (1:1000; PA5-18,039, lot # ZD4305091, Invitrogen) and rat anti-CD68 (1:1000; MCA1957, lot #1708, Bio-Rad). Rhodamine Red-X donkey anti-rat (1:1000; 712–295-150 Jackson ImmunoResearch) and Alexa Fluor 647 donkey anti-goat (1:1000; 705–605-147, Jackson ImmunoResearch). To label astrocytes, we used a rat-GFAP (Glial Fibrillary Acidic Protein) antibody (1:2500, 13–0300, lot #T1275011, Invitrogen) and Rhodamine Red-X donkey anti-rat (1:1000; 712–295-150 Jackson ImmunoResearch).

### Image analysis

All measurements were performed by experimenters blinded to treatment conditions using ImageJ (NIH) software. For each animal about 4 brain sections were analyzed and averaged. Regions of interest (ROIs) were drawn on the right hemisphere around the retrosplenial cortex (Rsp Ctx), which is one of the first areas to show functional impairment in AD and plays a key role in memory and spatial learning [45]; the area around the entorhinal cortex (Ent Ctx) which is often the earliest area to show histological alterations in AD and is involved in processing spatial and non-spatial information [46]; and around the hippocampus (Hipc), specifically the cornu ammonis regions 1 (CA1) and 3 (CA3) areas which are is known to be severely affected in AD and are essential for memory and spatial navigation [47].

Iba1 and CD68 positive cells were counted using ImageJ (NIH) software and classified based on ramified morphology and CD68 expression as described previously [37, 48, 49]. For astrocytes, in addition to the area covered, GFAP fluorescent staining intensity (IntDen) was measured in all ROIs using the raw unmodified/non- thresholded images, and GFAP positive cells were counted. For the CA1 and CA3 regions of the hippocampus, an r-score for astrocyte reactivity was calculated for each animal as described in Luijerink et al. [50]. The average score of two independent experimenters that were blinded to treatment group was used for statistical analysis. R-score could not be calculated in the cortical areas as cortical astrocytes express very low, even undetectable, levels GFAP under physiological conditions and only significantly increases only under reactive or pathological conditions [51–54]. Outliers were identified using Grubb’s test and excluded if affecting normality. For GFAP in the IntDen in the entorhinal cortex 1 mouse in the Intact VCID group, for the %area in the CA1: 1 mouse in the OVX VCID group; for the entorhinal cortex: 1 mouse in the Intact Sham and 1 in the Intact VCID group.

### Statistical analysis

Statistical analyses were completed using GraphPad Prism (GraphPad Software, San Diego, CA, USA). Data were evaluated for normal distribution and statistical outliers were identified using Grubbs’ test and removed if they affected normality which is indicated in the methods above. Data were analyzed using a 2-way ANOVA followed by Tukey’s post hoc test or 3-way repeated measures ANOVAs were used for measure tracked over time. Statistical significance was set at p < 0.05. Data are expressed as mean + SEM. Only main effects are presented on graphs unless an interaction effect is observed.

## Results

### Loss of ovarian hormones impairs cognition in both AD and MED models

**Fig. 2:**
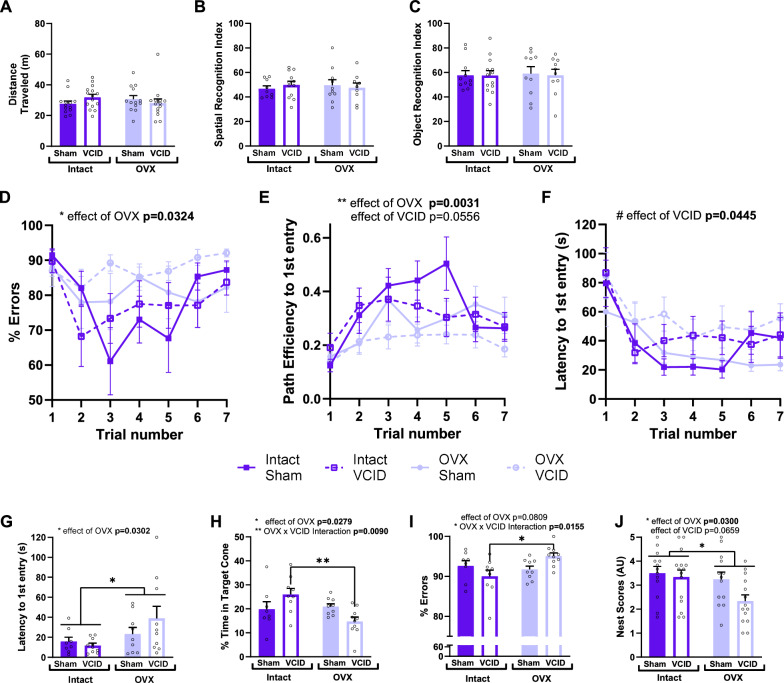
Ovariectomy and VCID affect cognitive behavior. Total distance traveled (m) in the open field (**A**) was measured as a proxy for locomotor activity. spatial recognition (**B**) and object recognition (**C**) indexes were obtained during the novel object place and the novel object recognition test respectively. data were analyzed using a 2-way ANOVA. n = 9–15 mice/group. OVX: ovariectomy, VCID: vascular contributions to cognitive impairment and dementia. spatial learning and memory were assessed using the barnes maze test (**D-I**). during the training trials (learning phase of the test), the percentage of errors/incorrect entries (**D**), the path efficiency for first entry (finding the escape hole, **E**), and the latency to first entry (**F**) were used to evaluate spatial learning. data were analyzed using a repeated measure 3-way ANOVA. * p < 0.05 main effect of VCID; * p < 0.05, **p < 0.01 main effect of OVX; n = 13–15 mice/group. memory was tested after a full day break during the probe trial by measuring the latency to first entry (**G**), the percentage of time spent in the target cone formed by the area encompassing 2 holes on each side of the target (**H**), and the percentage of errors (**I**). data were analyzed using a 2-way ANOVA with tukey’s multiple comparison test. * p < 0.05, **p < 0.01 main effect of OVX; n = 8–10 mice/group. The nest building test was used as an indicator of activities of daily living (**J**). data were analyzed using a 2-way ANOVA. * p < 0.05, main effect of OVX n = 13–15 mice/group. OVX: ovariectomy, VCID: vascular contributions to cognitive impairment and dementia

We have previously shown that loss of estrogens in a mouse model of menopause leads to exacerbated cognitive decline caused by VCID in WT female mice [35]. We sought to test whether this also occurs in AD and MED mouse models. We used App^NL−F^ mice that were either gonadally intact controls or OVX. Mice also received either sham surgery (AD only) or VCID-inducing surgery (MED). One month later, mice underwent a battery of behavioral tests (Fig. 1A). We validated our MED model by assessing decreased blood flow in the right side of the brain at the end of the experimental timeline. Using laser speckle imaging, we found that the VCID inducing surgery led to a sustained decrease in blood flow regardless of OVX ~ 7 weeks post-surgery (Fig. 1B, main effect of VCID p = 0.0376, F (1, 33) = 4.691). We next assessed the behavioral effects of OVX in both dementia models. We did not find any significant differences in locomotor activity using the open field test (Fig. 2A), or in spatial recognition memory using the novel object place test (Fig. 2B), or in object recognition memory using the novel object recognition test (Fig. 2C). However, we found changes in spatial learning and memory as tested by the Barnes maze test (Fig. 2D-I). We found that the loss of ovarian hormones led to impaired spatial learning, as evidenced by OVX mice having a higher number of errors (Fig. 2D, main effect of OVX p = 0.0324, F (1, 50) = 4.841), and a worse path efficiency to 1st entry (Fig. 2E, main effect of OVX p = 0.0031, F (1, 50) = 9.663). Cerebral hypoperfusion, resulting in MED, impaired spatial learning as well, which is supported by a longer latency of MED mice to find the escape hole (Fig. 2F, main effect of VCID p = 0.0446, F (1, 50) = 4.247) and a trend in having a worse path efficiency during the hidden trials (Fig. 2E, main effect of VCID p = 0.0556, F (1, 50) = 3.841). Spatial memory was impaired by loss of ovarian hormones as evident by a main effect of OVX during the probe trial of Barnes maze on latency to first entry (Fig. 2G, main effect of OVX p = 0.0302, F (1, 33) = 5.131), and the percentage of time spent in the target cone (Fig. 2H, main effect of OVX p = 0.0279, F (1, 32) = 5.306). Some OVX effects were dependent on dementia type as we found a significant OVX x VCID interaction on the percentage of time spent in the target cone (Fig. 2H, interaction p = 0.0090, F (1, 32) = 7.747) and the percentage of errors made throughout the probe trial (Fig. 2I, interaction p = 0.0155, F (1, 32) = 6.535). In both cases, MED OVX mice performed worse than AD OVX mice as indicated by Tukey’s post hoc test (Intact:VCID vs. OVX:VCID p = 0.0042 Fig. 2H and p = 0.0167 Fig. 2I respectively). The negative effects of the loss of ovarian hormones extended to the activities of daily living. This was supported by a main effect of OVX to reduce nest scores (Fig. 2J, main effect of OVX p = 0.0300, F (1, 51) = 4.985) in the nest building test (an assessment of activities of daily living). These results show that the loss of ovarian hormones impairs spatial learning and memory as well as activities of daily living in both AD and MED models.

### Loss of ovarian hormones or cerebral hypoperfusion increase β-amyloid

**Fig. 3:**
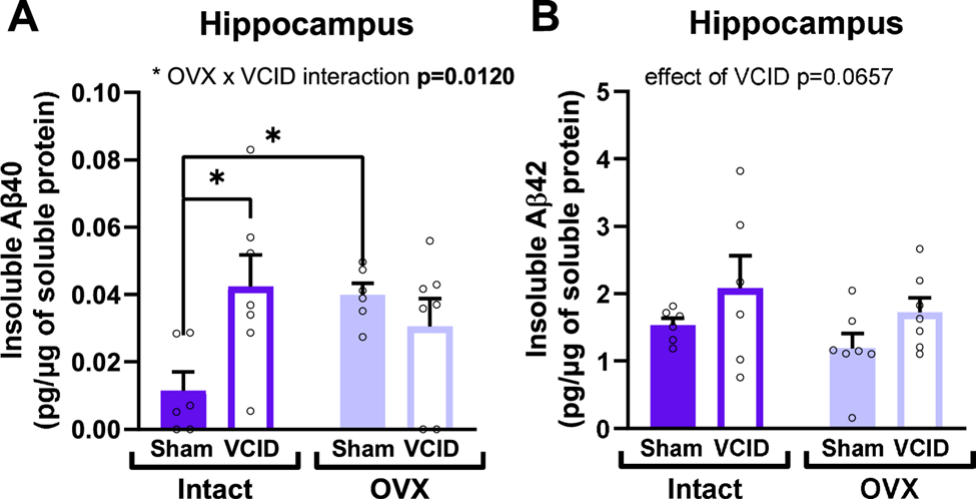
Ovariectomy or VCID increase insoluble Aβ peptides. ELISA quantification of human Aβ40 (**A**), and Aβ42 (**B**) in the insoluble fraction of proteins extracted from the hippocampus. A 2-way ANOVA was used followed by tukey’s multiple comparison test. * p < 0.05, **p < 0.01 n = 6–7 mice/group. OVX: ovariectomy, VCID: vascular contributions to cognitive impairment and dementia

Starting in early AD stages, insoluble β-amyloid peptides start to accumulate and agglomerate leading to plaque formation [55]. We used ELISA to quantify Aβ40 and Aβ42 peptides in the insoluble fraction of proteins extracted from the hippocampus and the cortex. In the hippocampus, we observed an OVX x VCID interaction [p = 0.0120, F (1, 22) = 7.506] wherein either VCID on its own compared to intact sham mice (Tukey’s post hoc test, Intact:Sham vs. Intact:VCID p = 0.0188), or OVX on its own compared to intact sham mice (Tukey’s post hoc test, Intact:Sham vs. OVX:Sham p = 0.0396) led to an increase in insoluble Aβ40 levels (Fig. 3A). For Aβ42 (Fig. 3B), we observed a trend in the main effect of cerebral hypoperfusion towards increased levels [main effect of VCID p = 0.0657, F (1, 22) = 3.751]. These results show that the loss of ovarian hormones seems to have selective effects on Aβ peptides.

### Loss of ovarian hormones increases astrogliosis in the CA3 of the hippocampus

**Fig. 4:**
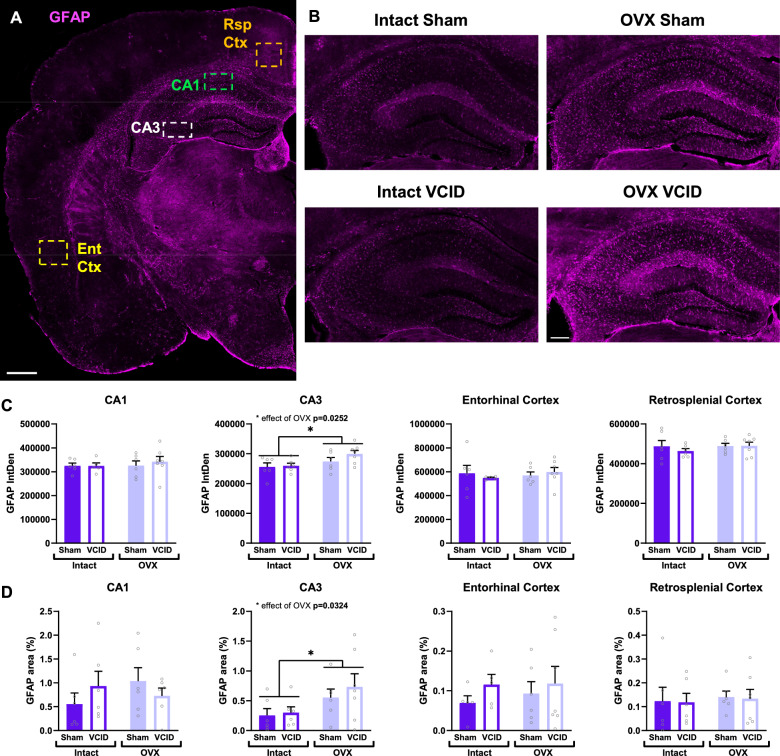
Ovariectomy leads to astrocyte reactivity in the hippocampus. Representative images of astrocyte immunofluorescent labeling using anti-GFAP (Glial Fibrillary Acidic Protein, magenta) are shown in a for placement of the ROIs, and in b for group comparisons. Scale bar in (**A**) 500 µm, and in (**B**) 200 µm. Quantification of the GFAP labeling intensity (IntDen), (**C**) and area density (%, **D**) in the CA1 and CA3 of the hippocampus, as well as in the restrosplenial and entorhinal cortices. A 2-way ANOVA and Tukey’s multiple comparison test were used to assess the effect of OVX and VCID; * p < 0.05 effect of OVX; n = 5–7 mice/group. OVX: ovariectomy, VCID: vascular contributions to cognitive impairment and Dementia, CA: cornu ammonis

In both AD patients and rodent models of AD, astrocytes can become reactive even before the formation of amyloid plaques [56–59]. Astrocyte reactivity, termed astrogliosis, is characterized by morphological changes (hypertrophy, increased volume and branching) and upregulation of GFAP expression [57, 58]. Using immunofluorescent labelling, we quantified the intensity (InDen) and coverage of the GFAP labeling to assess astrogliosis in several ROIs of the brain (Fig. 4A-B). We found an increase in both GFAP labeling intensity (IntDen) and area covered (%) in the CA3 region of the hippocampus of OVX mice regardless of dementia type [Fig. 4C, main effect of OVX p = 0.0252, F (1, 21) = 5.805, and Fig. 4D p = 0.0324, F (1, 21) = 5.248, respectively]. No changes were found in the other regions studied i.e. the CA1, EntCtx, and RspCtx (Fig. 4C-D). Changes in GFAP cell density and R-scores did not reach statistical significance levels in any region (Sup. Figure 1). The increases in GFAP labeling intensity and area indicate a potential increase in astrogliosis due to loss of ovarian hormones limited to the CA3 area.

### Loss of ovarian hormones and/or cerebral hypoperfusion impair microglial response

**Fig. 5:**
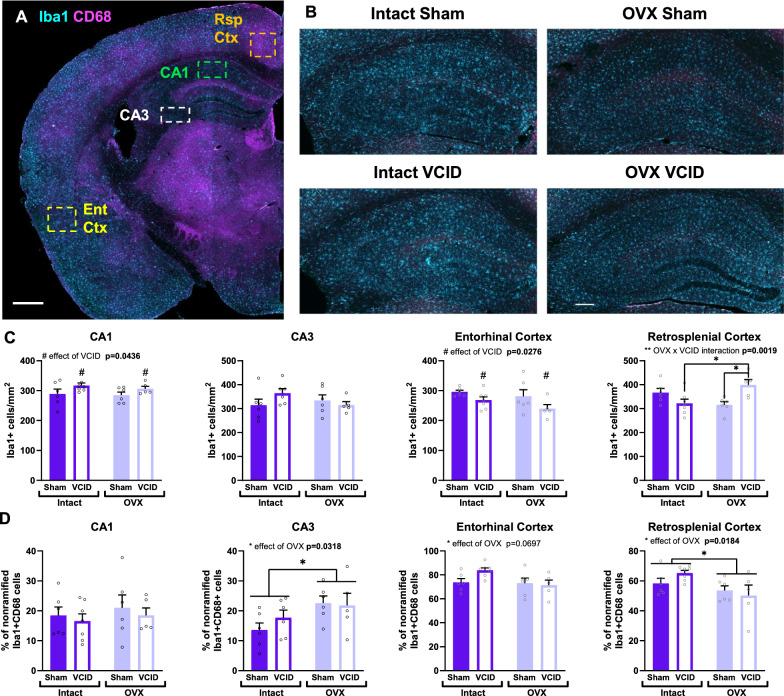
Ovariectomy and VCID impair microglia activation. Representative images of microglia labeling using anti-Iba1 (Cyan, microglia marker) and CD68 (magenta, lysosomal marker) are shown in a for placement of the ROIs, and in b for group comparisons. Scale bar in (**A**) 500 µm, and in (**B**) 200 µm. quantification of the number of Iba1 positive cells/mm^2^ (**C**), and the % of non-ramified Iba1 and CD68 positive cells (%) (**D**) in the CA1 and CA3 of the hippocampus as well as in the restrosplenial and entorhinal cortices. A 2-way ANOVA followed by Tukey’s multiple comparison test were used. # p < 0.05 effect of VCID; * p < 0.05, **p < 0.01; n = 5–7 mice/group. OVX: ovariectomy, VCID: vascular contributions to cognitive impairment and dementia, CA: cornu ammonis

Microglia, a major part of the innate immune system of the CNS, are emerging as potential therapeutic targets for AD [60, 61]. Findings using AD mouse models show that microglia reactivity in the early stages of disease is a complex process that can elicit both protective and detrimental effects [61–65]. We assessed microglia reactivity in the CA1 and CA3 of the hippocampus, the entorhinal cortex and the retrosplenial cortex using immunolabeling for Iba1 and CD68 (Fig. 5A-B). In the CA1, VCID led to an increase in Iba1 positive cell density [Fig. 5C CA1, main effect of VCID, p = 0.0436, F (1, 20) = 4.640]; no changes were observed in the percentage of activated microglia (non-ramified Iba1 +/CD68 + cells, Fig. 5D CA1). In the CA3, we did not detect changes in Iba1 positive cell density (Fig. 5C CA3); however, OVX led to an increase in the percentage of activated microglia [Fig. 5D CA3, main effect of OVX, p = 0.0318, F (1, 19) = 5.373]. In the EntCtx, VCID led to a decrease in Iba1 positive cell density [Fig. 5C EntCtx, main effect of VCID, p-0.0276, F (1, 20) = 5.646]; OVX led to a trend in decrease in the percentage of activated microglia (Fig. 5D EntCtx, effect of OVX, p = 0.0697). In the RspCtx, we observed an interaction in the effect of the two variables on Iba1 cell density [Fig. 5C RspCtx, OVX x VCID interaction, p = 0.0019, F (1, 20) = 12.85], wherein a combination of VCID and OVX led to a higher Iba1 cell density in the RspCtx of OVX:VCID animals compared to those that received OVX or VCID on their own (Tukey’s post hoc test, Intact:VCID vs. OVX:VCID p = 0.0301; OVX:Sham vs. OVX:VCID p = 0.0234). OVX led to a decrease in the percentage of activated microglia [Fig. 5D RspCtx, effect of OVX, p = 0.0184, F (1, 20) = 6.590]. All together these results indicate that in female App^NL−F^ mice, either cerebral hypoperfusion and/or loss of ovarian hormones lead to impaired microglia reactivity with regions specific effects.

## Discussion

Ovarian hormones, particularly E2, have several beneficial effects on the CNS [7, 9, 20–22, 66]. When women go through menopause, they suffer from a drastic reduction in hormone levels which increases their risk for AD and other dementias [11–18]. In this study, we sought to understand the effects of the loss of ovarian hormones on cognition and pathology in the most common dementia forms, AD and MED. We used the App^NL−F^ knock-in mouse model of AD and modeled MED by inducing cerebral hypoperfusion using a unilateral common carotid artery occlusion, a well-established VCID model. When comparing gonadally intact to ovariectomized mice, at a stage that corresponds to early disease pathology, we found that a loss of ovarian hormones leads to accelerated cognitive decline, increased Aβ levels, and impaired microglial response. Additionally, we observed that cerebral hypoperfusion had similar and sometimes additive effects on impairing learning and memory and exacerbating pathology. Our findings highlight the impact of ovarian hormones and emphasize the need for more inclusivity of sex-specific factors in preclinical dementia research. These data also suggest the need for considering other comorbidities, such as cerebral hypoperfusion, as covariates in dementia research and clinical settings.

Cognitive decline, a characteristic symptom of dementia, is known to be accelerated after menopause [9, 12, 67–70]. In this study, we show that, at early disease stages in the App^NL−F^ mice, the loss of ovarian hormones on its own compared to intact sham mice, was sufficient to induce deficits in spatial learning and memory as well as activities of daily living but not spatial recognition memory. A key difference between the OPRT and the Barnes maze test is the brain regions involved in behavioral outcomes. Even though both tests engage the CA1 dorsal hippocampus, only Barnes maze requires CA3 engagement, as it is important for spatial learning and allocentric spatial navigation [71–73]. Our histological analysis revealed changes in astrocytes and microglia that were significant in CA3 but not CA1. This points to CA3 being more affected than CA1 and therefore could explain the different results between behavioral tests. Further studies are needed to get to the underlying mechanism of these changes.

Behavioral deficits were observed in both AD and MED mice, though some were exacerbated by cerebral hypoperfusion in the MED mice. This is consistent with our previous study where menopause-induced loss of E2 in wild-type mice with cerebral hypoperfusion led to increased deficits in object recognition memory and activities of daily living [35]. However, in a recent study using the same AD mouse model, chemically induced menopause on its own was not sufficient to induce deficits in spatial learning and memory compared to intact sham mice [36]. This difference in results can be due to several factors, including menopause model (surgical vs chemically induced), the time after hormone loss, and the age and strain of the animals. For example, a study comparing the effect of OVX on cognition at different ages in wild-type mice found that the effects of OVX were greater in younger compared to older mice [74]. Moreover, in a study using the same spatial learning and memory test, the Barnes maze, OVX caused learning impairment in APP21 rats but not APP + PS1 rats (both rodent models of AD) [75]. A key difference between OVX and the chemically induced menopause model is that in the latter ovaries continue to secret androgens such as androstenedione and testosterone [76, 77]. Treatment of middle-aged (14mo) OVX female rats with high levels of androstenedione (8 mg/kg) impaired spatial memory versus vehicle or low dose (4 mg/kg) treated animals [78]. However, testosterone improved spatial memory in older (22mo) female mice [79]. Together, these findings highlight the complex interplay between ovarian hormones (types and levels), genetic background, and disease type and stage, underscoring the need for careful experimental design when assessing cognitive outcomes in dementia models.

E2 has been shown to reduce amyloid burden in vitro by promoting β-amyloid plaque degradation [80] and decreasing Aβ generation by primary cultures of rodent, and human embryonic neurons [81]. E2 treatment of ovariectomized non-human primates reduces the number of β-amyloid plaques [82]. In line with these results, we show that OVX led to an increase in the levels of insoluble Aβ peptides, the form that accumulates and agglomerates, leading to plaque formation. This further supports the beneficial role of ovarian hormones in limiting β-amyloid pathology.

Astrocyte reactivity is an important feature of AD and occurs early on in disease pathology before the formation of amyloid plaques [56–59, 83]. Our results show that loss of ovarian hormones increases GFAP labeling intensity and area covered (markers of astrocyte reactivity) in the CA3 region of the hippocampus. A recent study shows that loss of ovarian hormones in C57BL/6 J female mice led to a significant increase in reactive astrocytes and activated microglia in 12–14 months old animals in the dorsal CA1 area of the hippocampus [84]. This effect, however, was not detected in OVX younger mice (3–4 months old) [84]. These results indicate that the effects of ovarian hormones on astrocytes are age-dependent, which potentially explains why we do not see increases in reactivity in other brain regions such as the CA1. Changes in astrocytes can extend beyond neuroinflammation to alter the glymphatic function. Evidence from human observational studies and preclinical models shows that loss of E2 leads to impaired glymphatic clearance via several mechanisms including aquaporin-4 expression and polarization, altered perivascular structure and reduced clearance efficiency [85, 86]**.** Glymphatic clearance, important for Aβ elimination, is impaired by aging or in AD models, producing reduced Aβ clearance and increased amyloid burden [87, 88]. Exploring the effects of ovarian hormone loss on astrocytes and the glymphatic system could lead to better understanding of the mechanism by which menopause can lead to worse AD pathology.

Microglial activation is reported in AD, VCID, and menopause [89–95]. E2 is known to modulate microgliosis in rodents [96, 97]. We found that both the loss of ovarian hormones and cerebral hypoperfusion (independently or in combination) altered microglia density (Iba1 + cells/mm^2^) or reactivity (% of non-ramified Iba1 +/CD68 + cells) in a region-specific manner. Cerebral hypoperfusion led to an increase in microglia in the CA1 and, when combined with OVX, in the RspCtx. However, in the EntCtx, VCID animals had lower microglia density. Loss of ovarian hormones led to an increase of activated microglia in the CA3, but a decrease in the cortical areas. Several reasons can explain the surprising nature of these results; the first being that most studies on neuroinflammation are conducted exclusively in males. We had previously shown that in a mouse model of VCID with comorbid metabolic disease, microglial activation increased in the hippocampus of male but not female mice [49]. Other studies have also shown that female C57BL/6 J mice during aging and after a neuroinflammation challenge showed fewer activated microglia using similar immunolabeling markers [97]. Second, these differences can be due to the different animal models used. Previous results, from our lab and others, show that menopause leads to increased microglial activation in the hippocampus of the 5xFAD and 3xTg-AD transgenic mouse models that show a more aggressive pathology earlier in life [36, 98]. Third, the difference could emerge from the methods used to induce loss of ovarian hormones. In this study, we induced an abrupt cessation in hormone levels by OVX, whereas in the other studies mice went through a perimenopausal transition period and ovaries were left intact. This distinction is important, as in the chemically induced menopause model ovaries continue to produce other hormones which can have different effects in the brain, including on microglial activation [99–101]. Fourth, our analyses were performed at a single time point, and thus important acute or more long-term changes could have been missed. Finally, morphological changes in microglia do not necessarily correlate with transcriptional, proteomic, and functional profiles, which are better detected with different techniques such as single-cell and/or spatial transcriptomic analyses [102]. Another potential change in microglia could be their specific response to plaque clearance. Although not tested here, other studies have shown that microglial dysfunction, particularly failures in phagocytosis and plaque compaction, is a key driver of amyloid plaque accumulation [103]. E2 affects microglia activation and has been shown to enhance uptake of Aβ aggregates by human cortical microglia in vitro [104]. This suggests that loss of E2 after OVX can impair this mechanism. In addition, cerebral hypoperfusion is shown to cause microglia dysfunction in a mouse model of AD and cause a decrease of microglia phagocytic activity in low glucose in vitro environment mimicking that resulting from hypoperfusion [105]. Therefore, future and more detailed studies are needed to investigate microglia and neuroinflammation in female brains and how they are modulated by other factors.

## Conclusions

In summary, this study highlights the effects of ovarian hormones on multiple aspects of AD and MED including cognitive decline and histopathology. Some of these effects can be dependent on and exacerbated by cerebral hypoperfusion. This is of high importance, as only 3% of AD cases show pure AD pathology alone, while 82% had signs of MED [1, 106]. There is a need for additional studies at the preclinical and clinical stages which should consider comorbidities and sex-specific factors, as single-sex research -particularly in males- restricts our understanding and significantly impedes advancements in the elucidation of disease mechanisms and the discovery of effective therapies.

## Supplementary Information

Additional file 1.

## Data Availability

The datasets acquired and/or analyzed during the current study are available from the corresponding author on reasonable request.

## Abbreviations

E2: 17β-Estradiol
CA: Cornu ammonis region
Ent Ctx: Entorhinal cortex
GFAP: Glial fibrillary acidic protein
MED: Multi-etiology dementia
NORT: Novel object recognition test
OPRT: Object place recognition test
OVX: Ovariectomy
ROI: Regions of interest
Rsp Ctx: Restrosplenial cortex
UCCAO: Unilateral carotid artery occlusion surgery
VCID: Vascular contributions to cognitive impairment and dementia
