# Supplementary figures and images for: Loss of ovarian hormones is detrimental in early disease stages of mouse models of Alzheimer’s disease and multi-etiology dementia
Source: Biol Sex Differ. 2025 Dec 5;17:4. doi: 10.1186/s13293-025-00795-4 (PMC12797386; doi:10.1186/s13293-025-00795-4)

Sup. Figure 1: Changes in Astrocyte Numbers and Reactivity Score

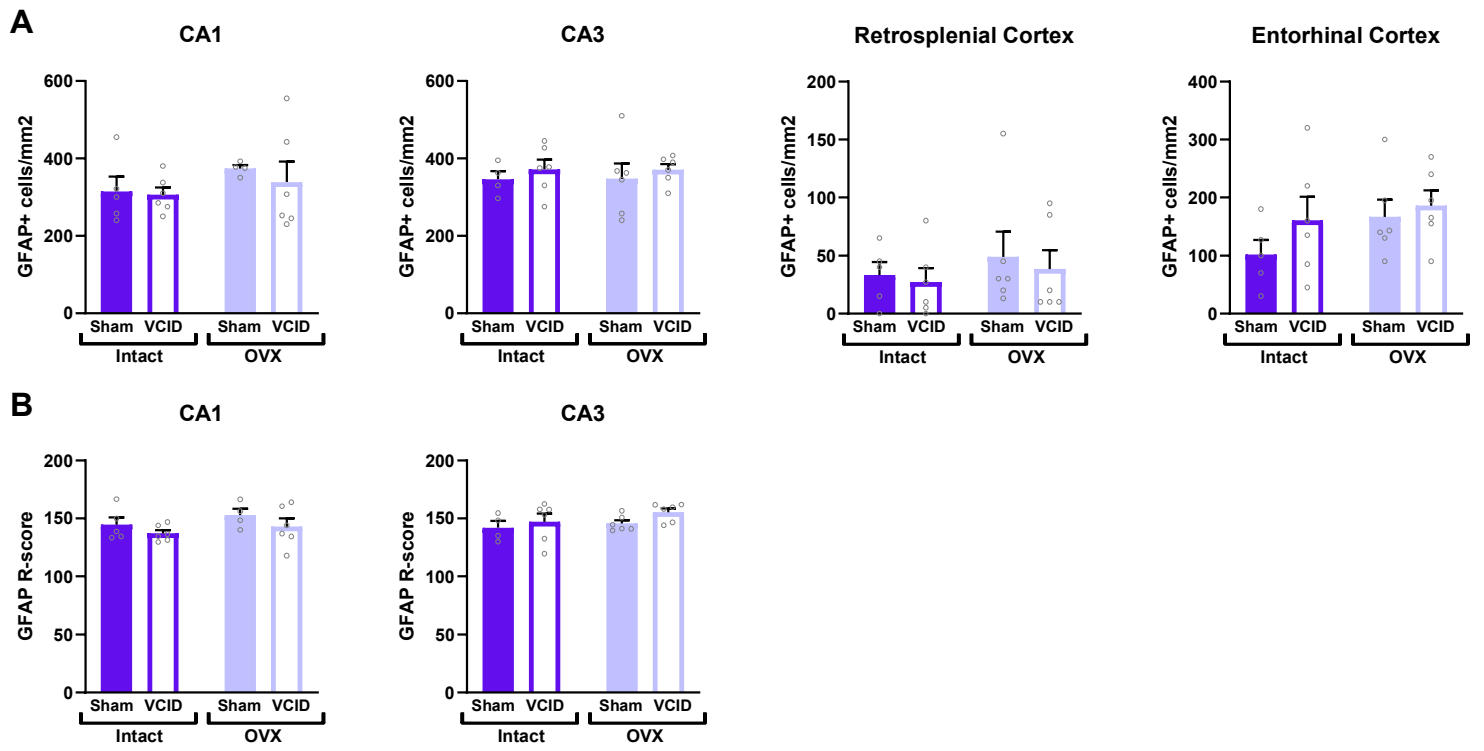

Supplement: Supplementary file 1 — Additional file 1. [file 13293_2025_795_MOESM1_ESM.pdf]
